# Epilogue: Lessons from the CONVERGE study of major depressive disorder in China

**DOI:** 10.1016/j.jad.2011.09.006

**Published:** 2012-09

**Authors:** Jonathan Flint, Yiping Chen, Shenxun Shi, Kenneth S. Kendler, CONVERGE consortium

**Affiliations:** a Wellcome Trust Centre for Human Genetics, Oxford OX3 7BN, UK; b Clinical Trial Service Unit, Richard Doll Building, Old Road Campus, Roosevelt Drive, Oxford OX3 7LF, UK; c Fudan University affiliated Huashan Hospital, No. 12 Wulumuqi Zhong Road, Shanghai 200040, PR China; d Shanghai Jiao Tong University School of Medicine affiliated Shanghai Mental Health Centre, No. 600 Wan Ping Nan Road, Shanghai 200030, PR China; e Virginia Commonwealth University, Department of Psychiatry, Virginia Institute for Psychiatric and Behavioral Genetics, Richmond, VA 23298-0126, United States

**Keywords:** Major depressive disorder, China, Co-morbidity, Anxiety

## Abstract

This review summarizes the first clinical reports from the CONVERGE consortium: China, Oxford and VCU Experimental Research on Genetic Epidemiology. CONVERGE sets out to investigate the nature and mode of action of the genetic and environmental risk factors for major depressive disorder (MDD). CONVERGE aims to collect 6000 cases of recurrent MDD and 6000 controls. The consortium includes hospitals in 30 cities, all with populations exceeding 5 million, and has collected over 2000 cases and controls. High quality phenotype data on MDD collected in China is now available to determine the source and nature of this highly heterogeneous condition. Analyses reported in a series of papers indicate that the clinical features and risk factors of MDD are sufficiently similar to those in the West that we can confidently predict that the results of subsequent analyses will be widely applicable.

The six articles in this issue of the JAD, together with five others submitted elsewhere, are the first fruits of an on-going three year collaboration between researchers in Oxford University in the UK, Virginia Commonwealth University in the US and 53 provincial mental health centers and psychiatric departments of general medical hospitals in the People's Republic of China. The principal aim of the CONVERGE consortium (China, Oxford and VCU Experimental Research on Genetic Epidemiology) is to investigate the nature and mode of action of the genetic and environmental risk factors for major depressive disorder (MDD), a disease projected to become the second leading cause of disability worldwide (after ischemic heart disease) by 2020 (Alonso et al., 2004; Demyttenaere et al., 2004; Melse et al., 2000; Michaud et al., 2001; Murray and Lopez, 1996; Ustun et al., 2004). The high prevalence, substantial economic costs and relatively ineffective performance of current treatments (Greenberg et al., 1993; Scott et al., 2003), mark out MDD as a research priority that demands concerted effort from many psychiatrists on a large scale.

In the absence of an established etiologic mechanism for MDD, a genetic approach that can interrogate the entire human genome is attractive because it requires no prior hypothesis about disease causation, other than the existence of a genetic contribution. Studies comparing concordance rates for MDD in monozygotic and dizygotic twins provide convincing evidence that the condition is indeed moderately heritable, with the best estimates of heritability of ~ 40% (Kendler et al., 2006a; Sullivan et al., 2000). Genome-wide association studies (GWAS) have now shown that it is possible to identify loci in the genome that contribute to the susceptibility to common, complex diseases, such as MDD (Lander, 2011). The molecular characterization of loci identified for other diseases is already leading to novel insights into etiological mechanism (Harismendy et al., 2011) and we expect the same to be true for depression once a sufficiently large and well characterized sample becomes available. CONVERGE's aim is to collect such a sample. However MDD poses a number of difficulties that are common in psychiatry, but less so for diseases that have been successfully subject to GWAS.

Multiple lines of evidence indicate that MDD is heterogeneous, both in its causes and in its symptomatology, and that it arises from the action and interaction between diverse environmental and genetic factors (Winokur, 1997). Discussions have gone on for over 100 years about the existence and number of subtypes of depression, each of which may reflect at least partly distinct diseases, with only modestly overlapping etiologic pathways. Environmental effects on MDD vary in their impact and mode of action, some acting on risk throughout the lifetime (Kendler et al., 2000a) others with much shorter risk periods (Kendler, 1998), suggesting that environmental factors operate via more than one pathway. There is also evidence that environmental effects impact differentially on different subtypes of MDD (Kendler et al., 2007).

Heterogeneity means that discovering, and investigating, risk factors for MDD requires a very big, well characterized clinical sample, much bigger than any previously collected, in which both biological and environmental effects can be jointly studied. Obtaining a sufficiently large and well-characterized sample is a key stumbling point in MDD research. To address this challenge, researchers in Oxford and VCU turned to their psychiatrist colleagues in China for help.

With funding first from the National Alliance for Research on Schizophrenia and Depression (now The Brain and Behavior Research Foundation) and then from the Wellcome Trust, CONVERGE grew to include hospitals in 30 cities, all with populations exceeding 5 million. The size of the hospitals, and their access to large numbers of patients, makes CONVERGE comparable to a collaboration between clinical services with responsibility for a population the size of Europe.

Six design features of the CONVERGE project are worthwhile summarizing here. First, CONVERGE studies only women because of evidence that the genetic risk factors for MDD are not entirely the same in men and women (Kendler et al., 2006a). Second, because of evidence that recurrence is the best index of a high familial/genetic loading for MDD (Sullivan et al., 2000), cases were required to have a history of at least two MDD episodes. Third, because of the relatively elevated base rates of MDD in the general population, controls are screened by personal interview to ensure that they had not experienced a prior depressive episode. Fourth, to maximize the genetic homogeneity of the sample, all four grandparents of both cases and controls are Han Chinese. Fifth, to reduce the probability that cases with recurrent MDD would not go on to develop bipolar disorder (which was an exclusion criteria in our study), a minimum age for cases is set at 30. Sixth, to reduce the probability that controls might go on to develop MDD, the minimum age for controls is 40.

In its relatively brief existence, CONVERGE has developed a bilingual (Mandarin–English) computerized interview, trained more than 100 doctors to conduct detailed research interviews, and recruited a smaller team to edit and review the data including audiotapes of all interviews. Key to CONVERGE's success has been effective communication and an ethos of scientific commitment in which all partners are equal contributors, all involved in the analysis and publication of results. This was achieved in three ways. First, the UK and US principal investigators travel to China to hold week long training sessions in the use of a computerized assessment system, designed in collaboration with Chinese psychiatrists. So far, three such meetings have taken place, in the cities of Shanghai, Hangzhou and Dalian, together with one refresher and problem-solving meeting in Shanghai. Second, the UK and Chinese principal investigators regularly visit participating hospitals, to identify and solve problems in ascertaining and assessing patients and controls, as well as to smooth out any unexpected difficulties that arise subsequent to the training sessions. Finally, the UK and US principal investigators have run one workshop in the analysis and presentation of results.

The workshop was held in the seaside city of Qingdao in the summer of 2010 with the ambitious aim of analyzing and writing up results from the first 2000 patients and 2000 controls that CONVERGE had by that date amassed. 60 doctors attended for three days. The results of their labors can be seen in the six articles in this issue of JAD, and the five published elsewhere. The first authors on these papers are young Chinese psychiatrists trained within the auspices of CONVERGE as interviewers, editors and now researchers. The emergence of this cohort of clinician–scientists is a major achievement of the international collaboration.

The story the articles tell is one of similarities and dissimilarities between MDD in China and the rest of the world. So far, our results suggest that the former far outweigh the latter. We see this as an important observation, and one that needs to put in the context of the difficulties associated with researching MDD in China. Kleinman has pointed out that “culturally coded symptoms may confound diagnosis” (Kleinman, 2004) and has written extensively about the difference in the ways mood is conceptualized and expressed in the middle kingdom (Kleinman, 1986, 2007). However, to date, no one had examined in depth the full range of clinical features and risk factors of MDD in a large Chinese sample.

Work from the CONVERGE project now shows that the major known risk factors for MDD operate in China just as they do elsewhere in the world. Data about the prevalence of stressful life events support the involvement of psychosocial adversity in the etiology of MDD in China, so that stressful life events have an impact comparable in magnitude to that seen in the West (Kendler et al., 2000c). CONVERGE was able to show that childhood sexual abuse substantially increases the lifetime risk of developing MD with the same kind of “dose–response” relationship seen in Western studies (Ferguson and Mullen, 1999; Kendler et al., 2000a). Similar to other studies in Western populations (Kendler et al., 2000b; Oakley-Browne et al., 1995; Rey, 1995; Rodgers, 1996a,b), high levels of a parenting style (authoritarianism) increase the risk for MDD, again with odds ratios very similar to those seen in a US sample (Kendler et al., 2000b).

The known biological risk factors operate just as they do in the West. Elevated levels of the personality trait neuroticism increase the risk for lifetime MDD. The effect of neuroticism on MDD is remarkably consistent across different countries: Western studies estimate the OR to be about 1.5 (Kendler et al., 2006b). CONVERGE's estimate is slightly less: 1.37. Familial factors are known to strongly influence the risk of developing MDD (Bland et al., 1986; Kendler and Prescott, 1999; Kupfer et al., 1989; Lyons et al., 1998; McGuffin et al., 1987, 1996), and they turn out to be equally important as a risk factor for MDD in China: CONVERGE finds that individuals with a high familial risk for MDD are characterized by severe episodes of MDD and have an earlier age of onset.

Clinical features, too, are consistent: Patients in the CONVERGE study with an earlier age of onset of MDD are more likely to suffer a chronic course, have higher comorbidity with anxiety and be less likely to marry. All of these features agree with findings from MDD patients ascertained in other parts of the world (Birmaher et al., 1996; Glied and Pine, 2002; Gollan et al., 2005; Klein et al., 1999; Lewinsohn et al., 1994; Parker et al., 2003; Zisook et al., 2004, 2007).

CONVERGE selected patients with recurrent MDD, usually hospitalized, so it is perhaps not surprising that the majority of cases have severe disease. Those with melancholia, making up 80% of cases, have a pattern of symptom severity, episode duration and comorbidity that echoes the extant literature (Angst et al., 2007; Joyce et al., 2002; Kendler, 1997; Rush and Weissenburger, 1994). Similarly, patients with dysthymia have a clinical picture broadly consistent with what has been seen in Western studies: greater comorbidity (Markowitz et al., 1992) and increased rates of a family history of MDD (Klein et al., 1988, 1995; Riso et al., 1996). CONVERGE has also found that the clinical features of post partum depression are very similar to those observed in the West, with a similar prevalence rate (Lee et al., 2001; O'Hara and Swain, 1996). As in the West, stressful life events increase the risk of post partum depression (Beck, 1996), and the same social (Bolton et al., 1998; Marks et al., 1992; O'Hara et al., 1991; Payne et al., 2009), and personality risk factors operate (Boyce et al., 1991; Saisto et al., 2001; Verkerk et al., 2005).

But there are dissimilarities too. Findings from CONVERGE are inconsistent with the simple hypothesis, from some European and US reports, that low levels of educational attainment increase the risk and severity of MDD (Alonso et al., 2004). In CONVERGE, subjects with more years of education are more likely to have MDD. Furthermore not all risk factors work as they do in the West. High parental protectiveness, which in Western countries generally increases the risk for MDD (Kendler et al., 2000b), is protective in China, especially when received from the father. CONVERGE also finds much lower levels of neuroticism in our controls than have been typically reported in Western samples (a mean of only 3.6, compared to European averages of 12 (Martin et al., 2000)).

This last example raises the important issue of the extent to which we can make meaningful cross-cultural comparisons when so much depends on the accuracy of translation between English and Chinese, and all results pass through a cultural filter, in which expectations and prior assumptions may not be identical between the two cultures. As Kleinman points out, “the concept of nonpsychotic depression is not indigenous among ordinary Chinese” and there is not a concept of mood disorder in traditional Chinese medicine (Kleinman, 2007).

Two important points need to be born in mind here. One is that the instruments CONVERGE deploys are developed in the West, so raising a question as to whether they can only see what they were designed to see. Taking personality as an example, the development and use of indigenous personality inventories, such as the Chinese Personality Assessment Inventory (Cheung et al., 1996), has led some to argue that a different personality model (six-factor model) is superior to the model commonly found in the West (five factor model) (Cheung et al., 2001). Second, cultural variation may simply reflect methodological differences between studies; personality is again a good example (Geisinger, 1994; van de Vijver and Leung, 1997): the context in which a test is administered may bias responses. Even though subjects fill in the personality questionnaires themselves, as is done Western studies, in CONVERGE they do so in front of a doctor. Their responses are likely affected by the respect Chinese traditionally hold for people in authority, such as doctors.

On the other hand, while there is an acknowledged risk that the instruments CONVERGE uses may lead us to miss, or worse misinterpret, clinical features, the sheer amount of information reveals new insights and opens up new avenues for exploration. For example CONVERGE was able to look at the relationship between education and MDD symptoms. This led to the surprising finding that lower educational attainment is associated with more neurovegetative symptoms and increased suicidal ideation and plans, while MDD patients with higher educational status more frequently experience hypersomnia and loss of interest. In short, education impacts differentially on the symptoms of depression. The rich dataset from CONVERGE has also allowed old ideas to be tested; there are some examples where CONVERGE results cast new light on received knowledge. In the work on post partum depression, the resemblance in presentation and risk factors contradicts an older literature that saw Chinese cultural practices protecting women from depression in pregnancy (Pillsbury, 1978; Stern and Kruckman, 1983).

CONVERGE is still in its infancy. The papers discussed here emerged from analysis of about one third of the projected sample size of 12,000 (due to be completed early in 2012). Three important lessons have already emerged. First, the clinical features and risk factors of MDD are sufficiently similar to those in the West that we can confidently predict that the results of subsequent analyses will be widely applicable. Second, as expected, high quality phenotype data on MDD can begin to detect the sources and nature of this highly heterogeneous condition. Third, as more data accumulate we expect new insights will emerge into psychiatry's most common disease.

CONVERGE is an example of how assaults on common disease require collaboration between doctors on a much larger scale than previously envisaged. This importance of size, of having a sufficiently large sample to detect the small effects, both genetic and environmental, is a lesson already learnt by physicians examining the genetics and phenomenology of cardiovascular (Newton-Cheh et al., 2009) and metabolic diseases (e.g. Voight et al., 2010). This lesson is if anything even more critical in psychiatry, where the problems of heterogeneity, disease definition and etiology are so much harder. CONVERGE has learnt that lesson and is beginning to harvest its reward.

## Role of funding source

Funding for this study was provided by the Wellcome Trust; the Wellcome Trust had no further role in study design; in the collection, analysis and interpretation of data; in the writing of the report; and in the decision to submit the paper for publication.

## Conflict of interest

All authors declare they have no conflicts of interest including any financial, personal or other relationships with other people or organizations within three years of beginning the work submitted that could inappropriately influence, or be perceived to influence, their work.
